# Supplementary material for: A Fit-for-Purpose Nutrient Profiling Model to Underpin Food and Nutrition Policies in South Africa
Source: Nutrients. 2021 Jul 28;13(8):2584. doi: 10.3390/nu13082584 (PMC8401225; doi:10.3390/nu13082584)
Supplement: Supplementary file 1 [file nutrients-13-02584-s001.zip › nutrients-1270510-supplementary.pdf]

Supplementary Table S1: Primary data collection methodology

Primary data collection methodology
Nutritional information on packaged food and beverages was collected through observation in supermarkets, to create a database of the nutritional content of packaged and processed foods available in the SA marketplace. In order to capture nutritional information on the product packaging photographs of all sides of all packaged food and beverage containers were taken in each store. At minimum, the bar code, package size, product name and NFP were captured.<br><br>To obtain a representative sample of packaged foods available in SA market stores were purposefully selected. Supermarkets in Cape Town (Western Cape) in Durbanville (at Pick 'n Pay, Woolworths, Checkers and Spar), Langa (Shoprite) and Khayelitsha (Boxer and Pick 'n Pay) were visited in February and March 2018. To ensure the variety of brands and products carried by different supermarkets were included, the four largest supermarket chains in SA where included. The stores together represented the majority of the grocery retailer market share, holding more than fifty percent of the share in SA in 2018 [1]. As supermarket stock in different socio-economic areas is likely to differ, different areas were sampled. As all packaged products were being explored it was necessary to include stores that carry a large product selection, which is the case in middle-income suburbs. Durbanville was included as the middle-class suburb, and Khayelitsha and Langa as the low-income suburbs.<br><br>A standardised protocol developed by The George Institute (TGI) was used to capture and submit in-store photographs (using cellphone cameras) of food labels during data collection. All packaged foods and beverages in the store at the time of data collection were included. Fieldworkers were university graduates, and trained in the data collection protocol. Nutrition information was captured using standardized methods and quality control measures by TGI appointed data capturers.<br><br>Foods and beverages were grouped into food categories for easier comparison. Conversion of foods and beverages requiring reconstitution (e.g. liquid concentrate beverages) from an "as sold" form to an "as consumed" form was done using information retrieved from product photographs when available. The raw dataset comprised of 9099 products, but after exclusion for insufficient and missing information (in SA a NFP is not required by law) the final dataset comprised of 6747 products (see figure S.1 for more information on excluded products). STATA (version 15, StataCorp, College Station, TX, USA) was used for data analyses and data cleaning. The nutrient content of products in the database were verified by identifying outliers and cross-checking against the original photographs of each product. Similarly missing nutrient information was verified, and corrected when possible.

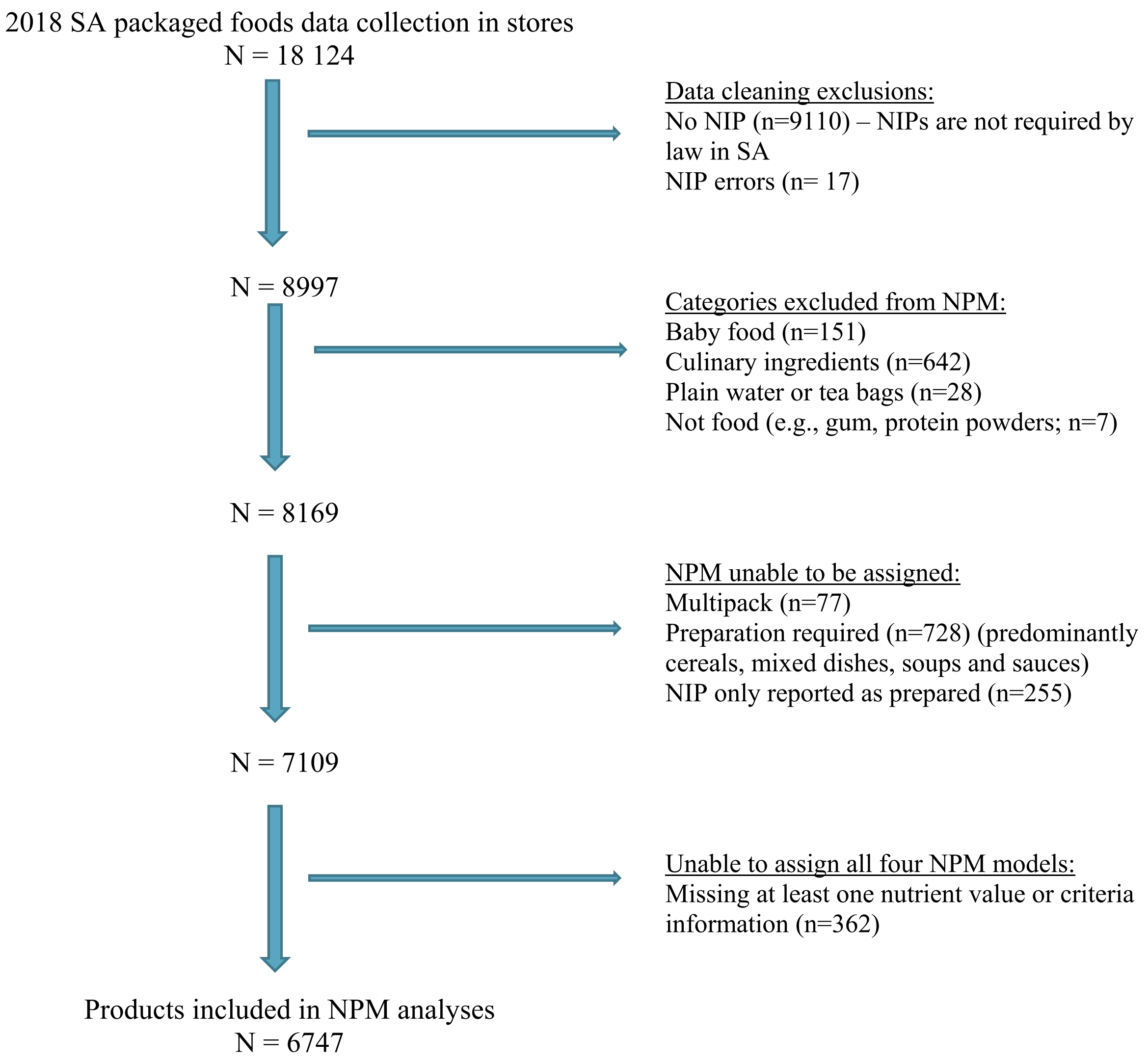

Supplementary Figure S1: Flow diagram representing initial and final datasets, and reasons for exclusion

Supplementary Table S2: Number and proportion of products included in analysis (N = 6747), overall and by food category

**FOOD**	**n**	**%**	**BEVERAGES**	**n**	**%**
Breakfast cereals	110	1.63	Dairy drinks	306	4.54
Cereals & cereal products	254	3.76	Other beverages	478	7.08
Confectionery & desserts	1119	16.59	Sodas	288	4.27
Dairy	791	11.72	100% fruit juice	385	5.71
Fruits	196	2.90	**Total**	**1457**	**21.59**
Vegetables	510	7.56			
Legumes	100	1.48			
Mixed dishes	299	4.43			
Protein	602	8.92			
Snack foods	699	10.36			
Soups & sauces	610	9.04			
**Total**	**5290**	**78.40**			

Supplementary Table S3: Non-sugar sweetener (NSS) search terms used to identify NSS ingredients in products

acesulfame	mannitol	sweet’n low	E-952
advantame	monatin	sweetnlow	E-953
alitame	monellin	sweetn low	E-954
altern	monk fruit extract	sweet nlow	E-955
aspartame	natrataste	sweet one	E-957
brazzein	natra taste	sweetone	E-959
candy leaf	nectasweet	syclamate	E-960
candyleaf	necta sweet	trichlorogalactosucrose	E-961
curculin	neohesperidine	twinsweet	E-962
cweet	dihydrochalcone	twin sweet	E-964
cyclamate	neotame	truvia	E-965
cyclamic acid	nutrasweet	thaumatin	E-966
enliten	nutra sweet	xylitol	E-967
acesulfame	osladin	E 420	E-968
equal	oubli	E 421	E-969
erythritol	pentadin	E 950	E420
galactitol	polyglycitol	E 951	E421
glucitol	purevia	E 952	E950
hydrogenated	reb a	E 953	E951
isomaltulose	reb-a	E 954	E952
instasweet	rebaudioside A	E 955	E953
insta sweet	rebiana	E 957	E954
isomalt	saccharin	E 959	E955
kaltame|	sorbitol	E 960	E957
lactitol|	splenda	E 961	E959
lumbah|	stevia	E 962	E960
luo han guo	steviol	E 964	E961
luohanguo	stevioside	E 965	E962
luohan guo	sucralose	E 966	E964
luo hanguo	sucrolase	E 967	E965
luo han kuo	sugar leaf	E 968	E966
luohankuo	sugarleaf	E 969	E967
luohan kuo	sugartwin	E-420	E968
luo hankuo	sugar twin	E-421	E969
mabinlin	sunett	E-950	
maltitol	sweetleaf	E-951	

Supplementary Table S4: Number and proportion of food and beverages (N = 6747) in the SA marketplace (2018), overall and by food category classified as ultra-processed according to the NOVA classification system

Food category	Number of products classified as minimally processed	Number of products classified as processed	Number of products classified as ultra- processed	% of processed / ultra-processed foods (according to NOVA classification)
Breakfast cereals n=110	12	1	97	89.09
Cereals & cereal products n = 254	28	11	215	88.98
Confectionery & dessert n = 1119	25	7	1087	97.77
Dairy n = 791	109	71	611	86.22
Fruits n = 196	72	37	87	63.27
Vegetables n = 510	141	96	273	72.35
Legumes n = 100	2	57	41	98.00
Mixed dishes n = 299	1	5	293	99.67
Protein n = 602	31	70	501	94.85
Snack foods n = 699	112	120	467	83.98
Soups & sauces n = 610	39	19	552	93.61
**FOOD TOTAL n = 5290**	**572**	**494**	**4224**	**89.19**
Dairy drinks n = 306	127	3	176	58.60
Other beverages n = 478	62	5	411	87.03
Sodas n = 288	1	2	285	99.65
100% juice n = 385	375	2	8	2.60
**BEVERAGE TOTAL n = 1457**	**565**	**12**	**880**	**61.22**
**FOOD & BEV TOTAL N = 6747**	**1137**	**506**	**5104**	**83.15**

Supplementary Table S5: Definitions of sugar and non-sugar sweetener used in this paper

**Term**	**Definition**
Total sugar	Includes intrinsic sugars, which are incorporated into the structure of intact fruit and vegetables; sugars from milk (lactose and galactose); and free sugars, which are monosaccharides and disaccharides added to food and beverages by the manufacturer, cook or consumer, and sugars naturally present in honey, syrups, fruit juices and fruit juice concentrates [2].
Added sugar	Monosaccharides or disaccharides added to food and does not include sugars naturally present in food, e.g. lactose in milk and fructose in fruit. Added sugar also includes honey [3].
Free sugar	Monosaccharides and disaccharides added to food and beverages by the manufacturer, cook or consumer and sugars naturally present in honey, syrups, fruit juice and fruit juice concentrates [2].
Non-sugar sweetener (NSS)	The definition used in the PAHO nutrient profiling model will be adopted: “Food additives that impart a sweet taste to a food, including artificial non-caloric sweeteners (e.g. aspartame, sucralose, saccharin and acesulfame potassium); natural non-caloric sweeteners (e.g. stevia); and caloric sweeteners such as polyols (e.g. sorbitol, mannitol, lactitol and isomalt). This does not include fruit juices, honey, or other food ingredients that can be used as a sweetener” [4].

Supplementary Table S6: Characteristics of nutrient profiling models considered for inclusion

	**Chile 2019** [5]	**Mexico 2020** [6]	**Peru 2020** [7]	**Israel 2020** [8]	**PAHO NPM 2016** [4]	**WHO African Region NPM 2019** [9]	**Food Standards Australia New Zealand (FSANZ) NPSC (updated 2016)** [10]
**Name of NPM**	Chilean warning octagons (CWO) 2016, 2018 and 2019 criteria	Mexico warning octagons 2020	Peru warning octagons 2020 (Part of Law on the Promotion of Healthy Diets)	Israel labelling 2020	Pan American Health Organization (PAHO) model	Nutrient profile model for the WHO African Region	Food Standards Australia New Zealand (FSANZ) Nutrient profiling score criteria (NPSC)<br>Current SA HNC NPM based on FSANZ NPM
**Country**	Chile	Mexico	Peru	Israel	Latin America and the Caribbean countries	African countries	Australia<br>New Zealand<br>South Africa
**Mandatory/ voluntary**	Mandatory	Mandatory	Mandatory	Mandatory	Voluntary	Voluntary	FSANZ NPSC: Mandatory if making claims
**Aim**	Restrictions on marketing to children under 14 years;<br>FOP warning label	FOP warning label	Restrictions on marketing to children under 14 years;<br>FOP warning label	FOP warning label	Restrictions on marketing and promotion to children; regulation in the school environment; FOP warning labels; application of taxes to limit consumption	Restrictions on marketing foods to children<br><br>Also: Used for tax policy to limit consumption of unhealthy foods, developing benchmarks for foods sold in public institutions; driver of reformulation, food labelling	For the regulation of health claims<br><br>Reformulation of products
**Rationale/ basis**	Implement the thresholds progressively in a period of 3 years from most permissive (June 2016) to more restrictive (June 2018) to final criteria (June 2019)	PAHO NPM used as a basis. Law and final regulations passed 27 March 2020	Guidance from PAHO, but final NPM very similar to Chile.<br><br>Implemented in 2 phases	Chile used as guideline	Based on WHO Population Nutrient Intake Goals (PNIGs); changes to the WHO PNIGs will be automatically incorporated into the PAHO NPM	Based on WHO PNIGs	Guiding consumers to the selection of foods consistent with the Australian and New Zealand Dietary Guidelines and developed with the collaboration of food industry.<br><br>Based on guideline daily amounts (GDA)<br>2000kCal for women

**Foods included by NPM**	Across the board – applies to all national/ imported packaged foods and beverages with added sugars, sodium or saturated fat	Across the board – applies to all national/ imported packaged foods and beverages with added free sugars, sodium or saturated fat	Across the board – applies to all national/ imported packaged foods and beverages with added sugars, sodium or saturated fat	Across the board – applies to all national/imported packaged foods and beverages with added sugars, sodium or saturated fat (excluding certain products – 1 ingredient foods)	Across the board for all processed and ultra-processed foods (based on NOVA classification)		Across the board, all foods included 3 food groups (beverages; cheese and fats; all other foods)
**Foods excluded by NPM (i.e. no cut offs)**	*Unpackaged foods<br>*Packaged foods with no added sugar, sodium or saturated fats	*Unpackaged foods; foods for medicinal purposes<br>*Dietary supplements<br>*Infant formula and follow-up milk	*Unpackaged foods<br>*Unclear what else	*Unpackaged foods<br>*Dietary supplements<br>*Infant formula<br>*Alcoholic beverages<br>*Specific products (tea, coffee)	*Unprocessed/min processed foods: vegetables, legumes, grains, fruits, nuts, roots and tubers, meat, fish, milk and eggs<br>*Freshly prepared dished, culinary ingredients (oils, sugar, honey, salt)<br>*Breast milk substitutes, food supplements, alcoholic beverages	*Special foods and supplements recommended for people with specific disease conditions<br>*Alcoholic drinks<br>*Breastmilk substitutes, including follow-up formula and growing-up milk	None
**Approach used in calculation/ cut-off used**	Threshold per nutrient	Threshold per nutrient	Threshold per nutrient	Threshold per nutrient	Threshold per nutrient	Category-based (18 categories and 10 sub-categories)	Scoring: final score determines whether a food is eligible to make a health claim
**Reference amount**	100g (solids) or 100ml (liquids)	Energy (kCal) and energy density	100g (solids) or 100ml (liquids)	100g (solids) or 100ml (liquids)	Energy (kCal)	100g (solids) or 100ml (liquids)<br><br>Except category 18 (sauces and dressings) – per serving	100g or 100ml
**Negative nutrient selection**	Energy<br>Saturated fat<br>Total sugar<br>Sodium	Energy<br>Saturated fat<br>Trans fat<br>Free sugar<br>Sodium<br>Non-sugar sweetener<br>Caffeine	Saturated fat<br>Trans fat<br>Total sugar<br>Sodium	Saturated fat<br>Total sugar<br>Sodium	Total fat<br>Saturated fats<br>Free sugar<br>Sodium<br>Any other sweetener<br>Trans fat	Energy<br>Total fat<br>Saturated fat<br>Total sugar<br>Added sugar<br>Sodium	Baseline points:<br>Energy<br>Saturated fats<br>Sugars<br>Sodium
**Positive nutrient selection**	No positive nutrients included	No positive nutrients included	No positive nutrients included	No positive nutrients included in mandatory FOPL.	No positive nutrients included	No positive nutrients included	Modifying points:<br>% fruits, vegetables, nuts and legumes (fvnl)

				However, voluntary positive FOPL is allowed for whole, unprocessed foodstuffs that do not carry an FOP warning label (and meet the voluntary positive FOPL criteria)[11].			Protein<br>Dietary fibre<br>Final score = Baseline points – modifying points<br>Can carry claim if score ≤4 (food); bev ≤ 1 and ≤28 (cheese & fats)

Bibliography:

1. Euromonitor International *South African Grocery Retailers Company Shares: Annual Report*; 2018;
2. World Health Organization *Guideline: Sugars intake for adults and children*; Geneva, 2015;
3. Medical Research Council *Food Composition Tables for South Africa*; 5th Editio.; Medical Research Council: Cape Town, South Africa, 2017;
4. Pan American Health Organization Nutrient Profile Model. *Nutr. Profile Model* **2016**, 1–32.
5. Food and Agriculture Organization; Pan American Health Organization *Approval of a new food act in Chile: Process Summary*; Santiago, 2017;
6. Secretaria de Economia Mexico Regulation NOM-051. **2020**, *1*, 4–40, doi:10.1017/CBO9781107415324.004.
7. Ministerio de Salud del Perú Aprueban Manual de Advertencias. **2018**, 58–63.
8. Ministry of Agriculture *Nutrition Labelling Regulation Israel: 7904*; Israel, 2017;
9. World Health Organization Regional Office for Africa *Nutrient Profile Model for the WHO African Region: a tool for implementing WHO recommendations on the marketing of foods and non-alcoholic beverages to childre*; Brazzaville, 2019;
10. Food Standards Australia New Zealand (FSANZ) *Nutrient profiling score criterion*; 2016;
11. Gillon-Keren, M.; Kaufman-Shriqui, V.; Goldsmith, R.; Safra, C.; Shai, I.; Fayman, G.; Berry, E.; Tirosh, A.; Dicker, D.; Froy, O.; et al. Development of Criteria for a Positive Front-of-Package Food Labeling: The Israeli Case. *Nutrients* **2020**, *12*, 1875, doi:10.3390/nu12061875.
